# BBX7 interacts with BBX8 to accelerate flowering in chrysanthemum

**DOI:** 10.1186/s43897-023-00055-2

**Published:** 2023-04-01

**Authors:** Yiwen Zhai, Yuqing Zhu, Qi Wang, Guohui Wang, Yao Yu, Lijun Wang, Tao Liu, Shenhui Liu, Qian Hu, Sumei Chen, Fadi Chen, Jiafu Jiang

**Affiliations:** National Key Laboratory of Crop Genetics & Germplasm Enhancement and Utilization, Key Laboratory of Landscaping, Ministry of Agriculture and Rural Affairs, Key Laboratory of Biology of Ornamental Plants in East China, National Forestry and Grassland Administration, Zhongshan Biological Breeding Laboratory, College of Horticulture, Nanjing Agricultural University, Nanjing, 210095 China

**Keywords:** BBX family, Diurnal rhythm, CmFTL1, Photoperiod, Flowering transition

## Abstract

The quantitative control of FLOWERING LOCUS T (FT) activation is important for the floral transition in flowering plants. However, the flowering regulation mechanisms in the day-neutral, summer-flowering chrysanthemum plant remain unclear. In this study, the chrysanthemum BBX7 homolog *CmBBX7* was isolated and its flowering function was identified. The expression of *CmBBX7* showed a diurnal rhythm and *CmBBX7* exhibited higher expression levels than *CmBBX8*. Overexpression of *CmBBX7* in transgenic chrysanthemum accelerated flowering, whereas lines transfected with a chimeric repressor (*pSRDX-CmBBX7*) exhibited delayed flowering. Yeast single hybridization, luciferase, electrophoretic mobility shift, and chromatin immunoprecipitation assays showed that CmBBX7 directly targets *CmFTL1*. In addition, we found that CmBBX7 and CmBBX8 interact to positively regulate the expression of *CmFTL1* through binding to its promoter. Collectively, these results highlight CmBBX7 as a key cooperator in the BBX8–FT module to control chrysanthemum flowering.

**Graphical Abstract:**

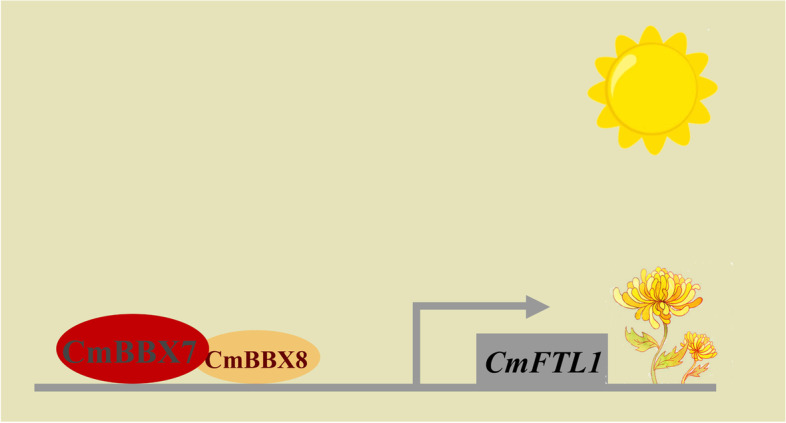

**Supplementary Information:**

The online version contains supplementary material available at 10.1186/s43897-023-00055-2.

## Core

*CmBBX7* shows a diurnal rhythm expression pattern and is expressed at higher levels than *CmBBX8*. CmBBX7 and CmBBX8 proteins interact to positively regulate the expression of *CmFTL1*, which controls flowering activation, through binding to its promoter.

### Gene and accession numbers

Sequence data from this article can be found in the database of the National Center for Biotechnology Information (NCBI) under the accession numbers: CmBBX7 (KP963937.1), CmBBX8 (KP963933.1), AtBBX1 (NM_121589.2), AtBBX2 (NM_121590.2), AtBBX3 (NM_111105.3), AtBBX4 (NM_128038), AtBBX5 (NM_122402.3), AtBBX6 (NM_125149.3), AtBBX7 (NM_111644.5), AtBBX8 (NM_124200.3), AtBBX9 (NM_001341021.1), AtBBX10 (NM_113084.2), AtBBX11 (NM_130356.5), AtBBX12 (NM_179880.2), AtBBX13 (NM_102570.4), AtBBX14 (NM_105523.3), AtBBX15 (NM_102355.5), AtBBX16 (NM_106047.5), AtBBX17 (NM_103803.4), AtBBX18 (NM_127704.3), AtBBX19 (NM_120056.6), AtBBX20 (NM_120067.7), AtBBX21 (NM_106207.4), AtBBX22 (NM_106507.4), AtBBX23 (NM_117092.3), AtBBX24 (NM_100484.4), AtBBX25 (NM_128695.4), AtBBX26 (NM_104715.1), AtBBX27 (NM_105490.4), AtBBX28 (NM_118865.3), AtBBX29 (NM_124827.5), AtBBX30 (NM_001036568.3), AtBBX31 (NM_113085.4), AtBBX32 (NM_113009.3), HaBBX7 (XP_021998233), HaBBX8 (XP_021989484), GmBBX7 (Glycine max, XP_003542186), AaBBX7 (Artemisia annua, PWA72942).

## Introduction

Flowering represents the transition from vegetative to reproductive growth and is regulated by multiple endogenous and exogenous cues. In the model plant Arabidopsis, the integrator FLOWERING LOCUS T (FT) in the leaves and SUPPRESSOR OF OVEREXPRESSION OF CONSTANS 1 (SOC1) integrate multiple flowering pathways (Lee and Lee 2010). The photoperiod plays a critical role in the flowering transition, and the relative time between light and darkness offers a cue for the precise regulation of the process so that the plant’s flowers only emerge under appropriate environmental conditions. In the photoperiod pathway, BBX1/CONSTANS (CO) is a transcriptional activator that directly regulates the expression of *FT* for flowering (Putterill et al. 1995; Song et al. 2015).

The BBX family of proteins is characterized by conserved B-box domains, with some members also having CCT domains (Khanna et al. 2009). According to the number of B-box domains and the presence or absence of the CCT domain, 32 BBX proteins have been identified and divided into five subgroups in Arabidopsis (Khanna et al. 2009; Gangappa and Botto 2014). *BBX1/CO* was the first BBX gene to be identified, which is regulated by the circadian clock (Suarez-Lopez et al. 2001), thereby directly activating the expression of *FT* (Wenkel et al. 2006; Cao et al. 2014). The CO-FT module performs central functions in the photoperiodic flowering pathway. Other members of the BBX family also have functions in flowering. For example, BBX4 negatively regulates flowering in Arabidopsis (Tripathi et al. 2017), which is consistent with the roles identified for BBX5 (Steinbach 2019), BBX7 (Cheng and Wang 2005), and BBX17 (Xu et al. 2022). However, BBX6 was shown to positively regulate flowering (Hassidim et al. 2009), and a similar function has also been identified for BBX24 (Li et al. 2014). Conversely, the chrysanthemum BBX24 homolog CmBBX24 was shown to play a negative role in flowering (Yang et al. 2014), indicating functional diversification of BBX among different plant species.

Interestingly, some members of the BBX family interact with one another to regulate flowering. For example, the N-terminus of BBX4 interacts with BBX32 and directly binds to the *FT* promoter through its CCT domain, thereby inhibiting its expression and delaying flowering (Tripathi et al. 2017). Similarly, BBX17 physically associates with CO to repress its role in activating *FT* transcripts (Xu et al. 2022). BBX19 also represses the role of CO in the regulation of flowering (Wang et al. 2014), whereas both BBX28 and BBX29 associate with CO to accelerate flowering (Wang et al. 2021a, b, c). In rose, *RcCO* and *RcCOL4* were identified as flowering promoters whose expression is upregulated under long-day (LD) and short-day (SD) conditions, respectively, and RcCOL4 interacted with RcCO to promote RcCO binding to the *RcFT* promoter, thereby activating its transcription (Lu et al. 2020).

We previously reported that the chrysanthemum BBX8 homolog *CmBBX8* targeted the *CmFTL1* promoter element (CORE) to induce its transcription, thereby promoting flowering in chrysanthemum (Wang et al. 2020). Although *AtBBX7*, a member of BBX subgroup II, has been shown to be involved in the negative regulation of flowering in *Arabidopsis thaliana* (Cheng and Wang 2005), the mechanism underlying the role of BBX7 in the regulation of chrysanthemum flowering remains unclear. In this study, we revealed that CmBBX7 directly interacts with CmBBX8 to enhance the transcriptional regulation of *CmFTL1* to accelerate flowering in chrysanthemum.

## Results

### Transcriptional expression analysis of *CmBBX7* in chrysanthemum cv. ‘Yuuka’

**Fig. 1:**
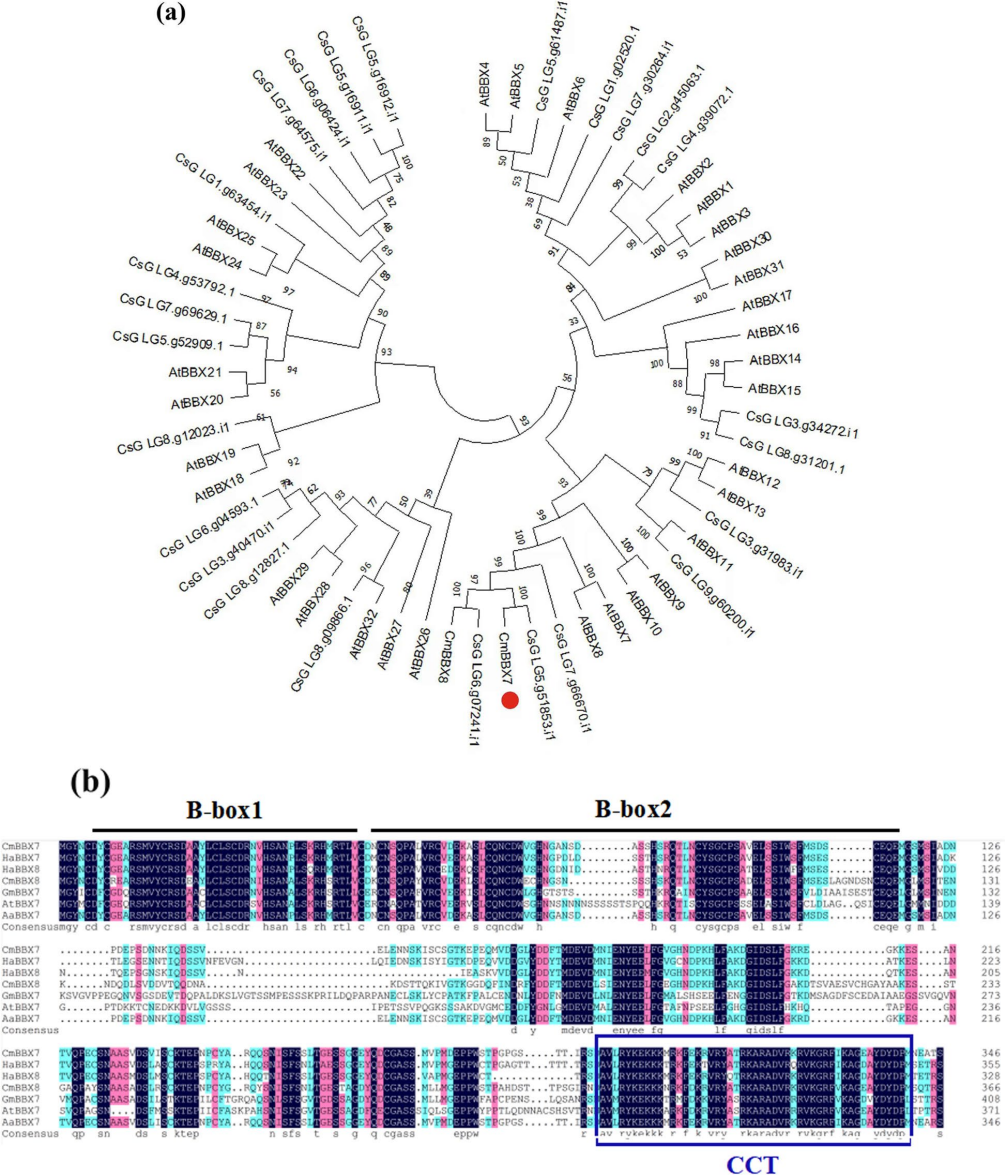
*CmBBX7* encodes a BBX family subgroup II protein. **a** Phylogenetic relationships of *CmBBX7* and *BBX* sequences from Arabidopsis and *Chrysanthemum seticuspe*. Protein sequences of the 32 BBXs in Arabidopsis were obtained from The Arabidopsis Information Resource (TAIR) database, the sequence of BBX8 in chrysanthemum was obtained from the National Center for Biotechnology Information (NCBI), and the genome sequence and annotation data of *C. seticuspe* are available at PlantGarden with Project ID PRJDB7468. The percentage of trees in which the associated taxa clustered together is shown next to the branches. **b** Alignment of the deduced polypeptide sequences of CmBBX7 with those of other plant BBXs. The B-box1 and B-box2 domains are indicated by the black lines above the sequences. The CCT motif is indicated by the blue lines around the sequences

To investigate the function of *BBX7* (CL7046.Contig5_All) in chrysanthemum, we isolated the *BBX7* sequence from chrysanthemum cv. ‘Yuuka’, which contains a 1044-bp open reading frame (ORF) encoding 347 polypeptide residues. Phylogenetic analysis showed that the isolated sequence had the highest homology with *AtBBX7* in Arabidopsis, and homologous genes were also identified in *Chrysanthemum seticuspe* (Fig. 1a); therefore, this gene was designated *CmBBX7*. The N-terminus of CmBBX7 includes two highly conserved B-box domains with a CCT domain at the C-terminus, which is a characteristic of subgroup II of the BBX family (Fig. 1b).

**Fig. 2:**
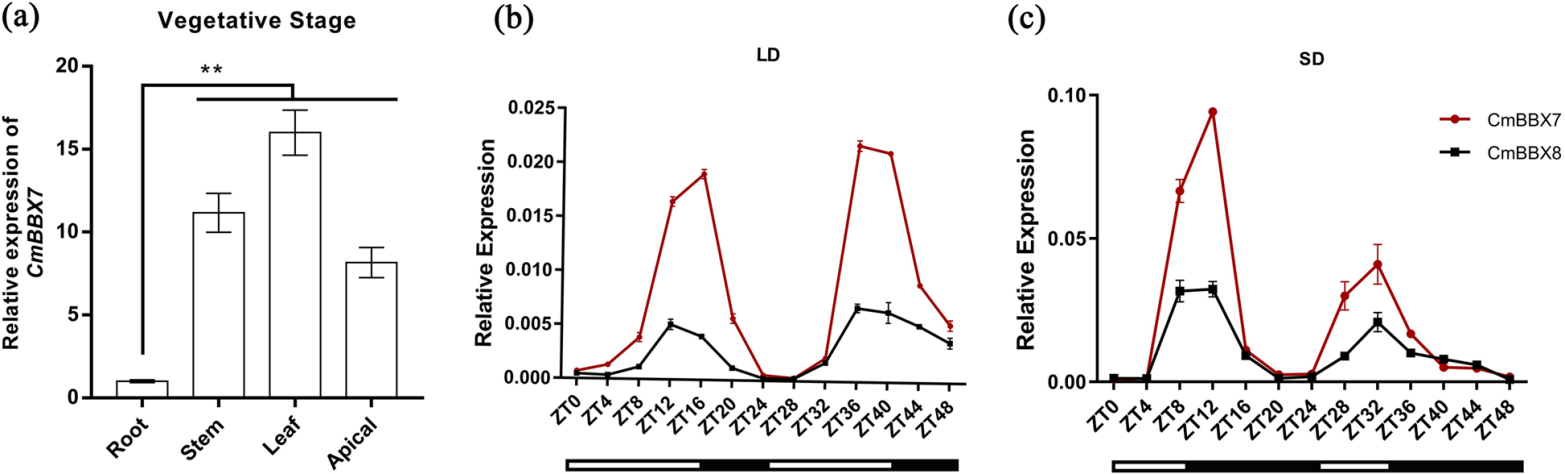
Transcriptional expression analysis of *CmBBX7* in chrysanthemum cv. ‘Yuuka’. **a** Relative mRNA expression levels of *CmBBX7* in various parts of the plant during the vegetative stage. Error bars indicate the standard errors for three biological replicates. Significant differences are indicated with asterisks (**p < 0.01, ANOVA with Tukey’s post-hoc test). **b**,**c** The transcriptional response of *CmBBX7*, and *CmBBX8* to varying photoperiods [long day (LD) and short day (SD)]. The abscissa indicates the sampling time point. Values shown are the means (*n* = 3) and the error bars represent the standard errors of the mean

We determined the expression levels of *CmBBX7* in the apical meristem, leaf, stem, and root during the vegetative stage. *CmBBX7* was transcribed in the apical meristems, leaves, stems, and roots, with the highest transcription abundance detected in the leaves (Fig. 2a). We further investigated whether the transcription of *CmBBX7* in the leaves is regulated by the diurnal rhythm. Under LD conditions (16-h light/8-h dark), *CmBBX7* expression levels showed oscillations, with a peak at approximately 16 h zeitgeber (ZT16), followed by a second peak at ZT36; under SD conditions, the peak was also detected at approximately ZT12, followed by a second peak at ZT32, which is consistent with that of *CmBBX8*. We previously found *CmBBX8* and *CmFTL1* showed overlapping expression in similar spatial–temporal patterns (Wang et al. 2020). Interestingly, *CmBBX7* had higher expression levels than *CmBBX8*, which indicated that *CmBBX7* plays an important role in the regulation of chrysanthemum flowering (Fig. 2b, c).

### *CmBBX7* accelerates the floral transition

**Fig. 3:**
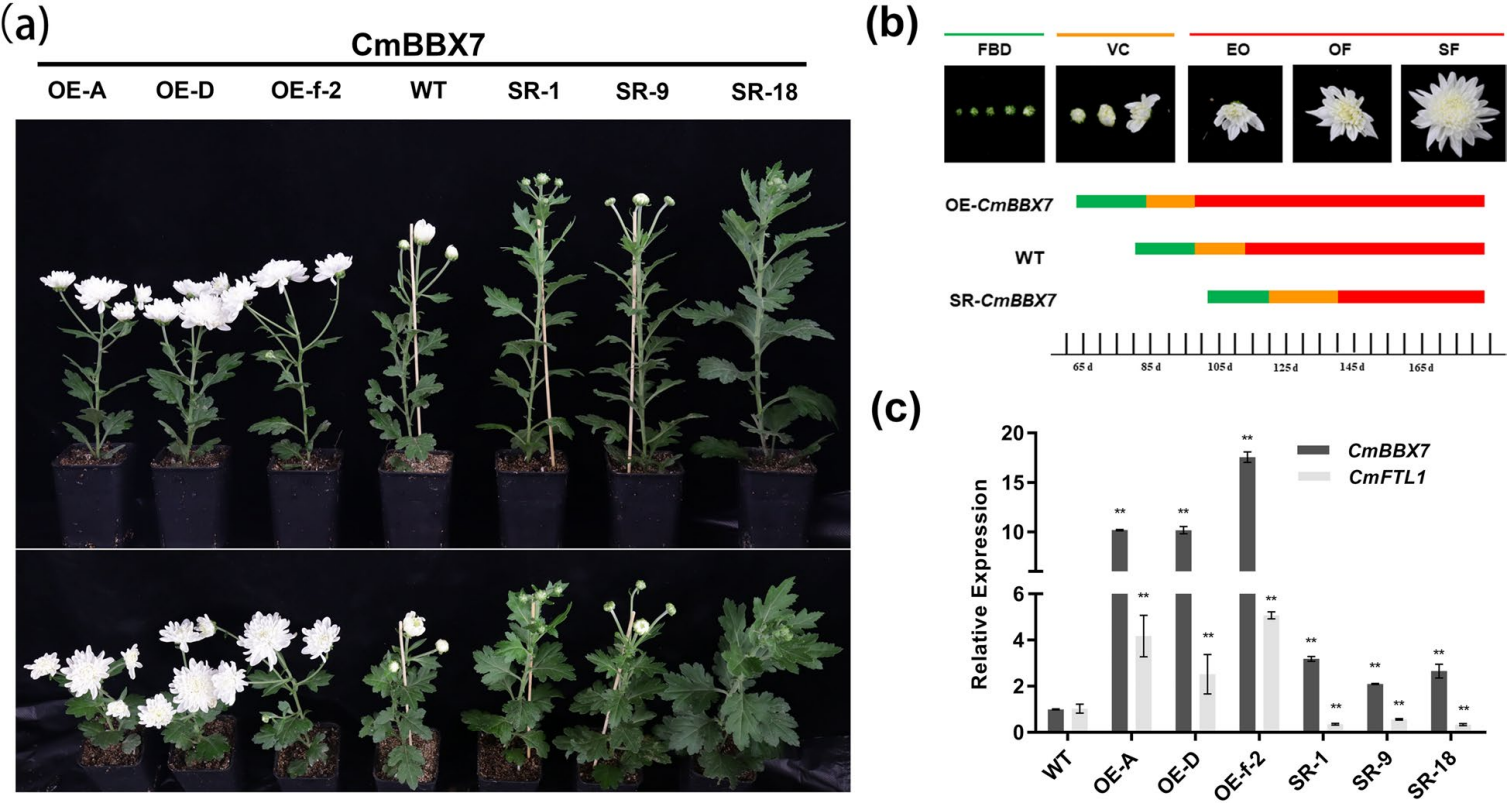
*CmBBX7* accelerates the floral transition. **a** The phenotypes of the *CmBBX7* transgenic lines and wild-type (WT) plants. The upper panel shows the elevation view and the lower panel shows the top view. OE: overexpression lines; SR: transcriptional repression lines. **b** Statistics of flowering time. FBD: flower bud development stage; VC: visible color stage; EO: early opening stage; OF: opened flower stage; SF: senescing flower stage. The lower bars show the time taken to grow the plants from planting to the various stages of flowering. **c** Relative expression levels of *CmBBX7* and *CmFTL1* in transgenic plants. Error bars indicate the standard deviations for three biological replicates. Significant differences are indicated with asterisks (**p < 0.01, ANOVA with Tukey’s post-hoc test)

To determine whether *CmBBX7* is involved in promoting flowering, a group of nine *CmBBX7* transgenic plants (OE-CmBBX7 plants) was obtained. Because we found that CmBBX7 had transactivation activity in yeast cells (Supplementary Fig. S1), a chimeric repressor construct (pSRDX-CmBBX7) was transformed into chrysanthemum, and five transgenic plants with inhibited transcriptional activation of *CmBBX7* were obtained (pSRDX-CmBBX7). Three lines were selected to evaluate the phenotypic characteristics of *CmBBX7*. According to statistics, OE-CmBBX7 plants began to enter the bud-formation stage 45 days after transplantation under LD conditions, whereas wild-type (WT) plants needed 60 days to reach this stage. In contrast, pSRDX-CmBBX7 plants reached the flowering stage 80 days after transplanting (Fig. 3a, b). *CmFTL1* expression was upregulated in OE-CmBBX7 plants and was downregulated in pSRDX-CmBBX7 plants (Fig. 3c).

### CmBBX7 directly regulates* CmFTL1*

**Fig. 4:**
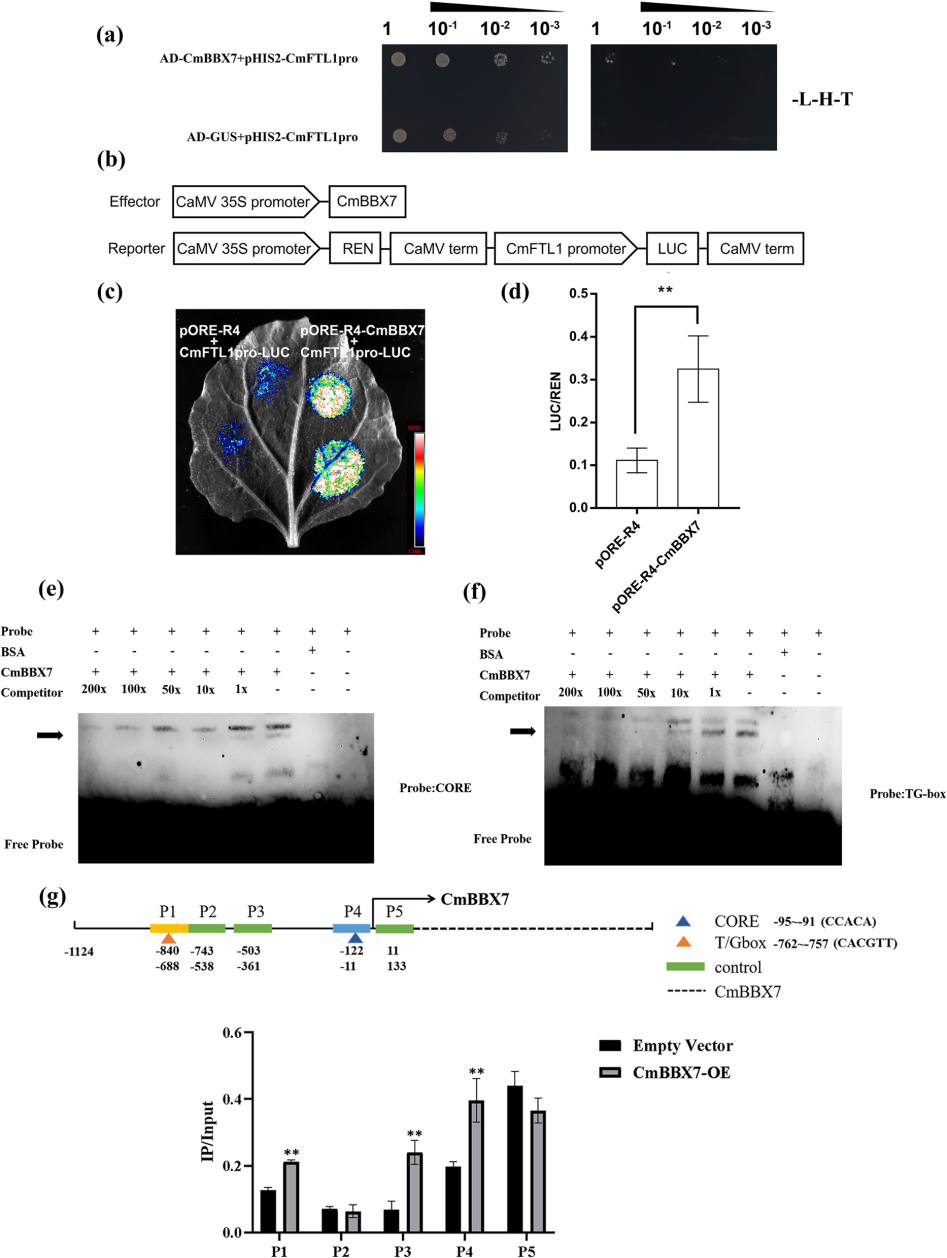
CmBBX7 regulates *CmFTL1* directly. Interactions of CmBBX7 protein with the promoters of *CmFTL1* in the yeast one-hybrid assay. SD/-T/-H/-L indicates Trp, His, and Leu synthetic dropout medium, respectively. The 3-AT concentration is 60 mM for *CmFTL1pro*. **b** Effector and reporter vector construction diagrams for the dual-luciferase assays. **c** Transient expression in *N. benthamiana* leaves of the *pCmFTL1::LUC* transgene. Graph showing luminescence intensity. **d** The ratio of LUC to REN activity. Error bars indicate the SD for six biological replicates. Significant differences are indicated by asterisks (** *p* < 0.01, ANOVA, Tukey’s correction). **e**–**f** EMSA of CmBBX7 binding to the CORE and TG-box elements of *CmFTL1* promoter. ‘ + ’ indicates presence and ‘ − ’ indicates absence. **g** ChIP-qPCR of the enrichment of DNA fragments CORE (–95 to –91 bp) and TG-box (–762 to –757 bp) in the *CmFTL1* promoter. Error bars indicate the SD for six biological replicates. Significant differences are indicated by asterisks (** *p* < 0.01, ANOVA, Tukey’s correction).

Considering that *CmBBX7* overexpression could promote the expression of *CmFTL1*, we hypothesized that CmBBX7 is directly involved in regulating *CmFTL1* expression by binding to its promoter. To confirm this hypothesis, the *CmFTL1* promoter sequence was constructed using a fusion pHIS2 vector for a yeast single hybridization (Y1H) assay. The strain grew normally on SD/-Trp/-His/-Leu medium supplemented with 3-amino-1, 2, 4-triazole (3-AT) only when pHIS2-CmFTL1pro was co-expressed with pGAD7-CmBBX7 in yeast (Fig. 4a). Next, the *CmFTL1* promoter sequence was fused with the luciferase (*LUC*) gene as a reporter and used to perform dual-luciferase assays in *Nicotiana benthamiana* leaves. The results confirmed that CmBBX7 significantly enhanced the ability to activate *CmFTL1-LUC* (Fig. 4b,c). Chrysanthemum protoplast transfection experiments confirmed that overexpression of *CmBBX7* significantly increased the LUC/*Renilla* luciferase (REN) activity ratio compared with that of the empty control (Fig. 4d). Furthermore, the electrophoretic mobility shift assay (EMSA) demonstrated that CmBBX7 could combine the CORE (CCACA, –95 to –91 bp) and TG-box (CACGTT, –762 to –757 bp) elements in the *CmFTL1* promoter in vitro (Fig. 4e, f). To further support this conclusion, we performed a chromatin immunoprecipitation (ChIP)-quantitative polymerase chain reaction (qPCR) assay, which clearly showed that compared with the control (P2 and P5 fragments), P1, P3, and P4 fragments presented enrichment, proving that CmBBX7 can specifically recognize the CORE, TG-box, and other unknown elements in the *CmFTL1* promoter (Fig. 4g).

### CmBBX7 interacts with CmBBX8

**Fig. 5:**
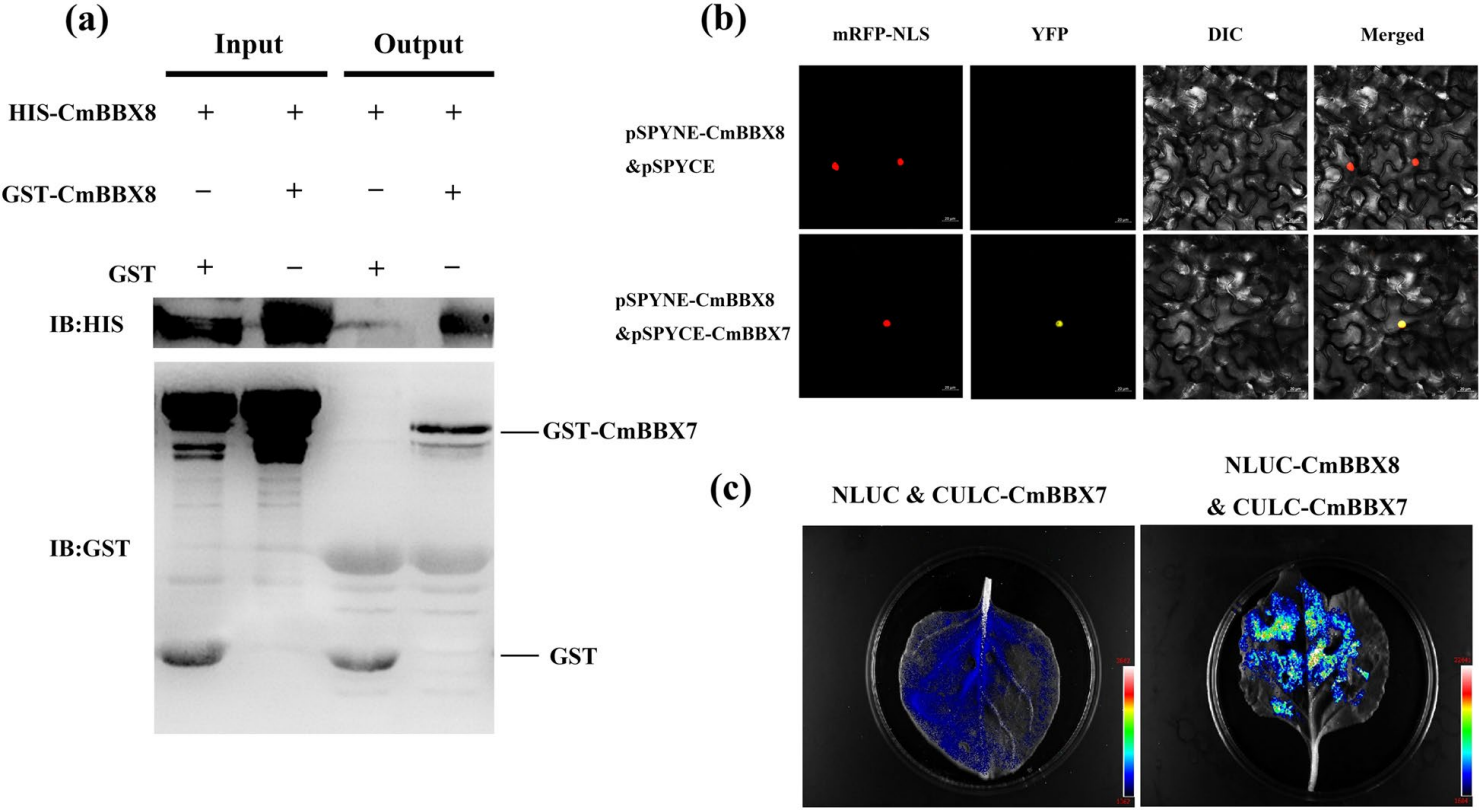
CmBBX7 interacts with CmBBX8. **a** Pull-down assays of the interaction between CmBBX7 and CmBBX8. The arrows show the target protein sizes. GST-empty vector and His-CmBBX8 were used as controls. **b** BiFC assay. mRFP-NLS: images taken in the red fluorescence channel; YFP: images taken in the yellow fluorescence channel; DIC: images taken in bright light; Merged: both overlay plots; bars = 20 μm. **c** Firefly luciferase complementation imaging (LCI) assay verified the interaction between CmBBX7 and CmBBX8 in *N. benthamiana* leaves

In our previous report, we showed that CmBBX8 could directly activate the transcript of *CmFTL1* (Wang et al. 2020); thus, we speculated that CmBBX7 cooperates with CmBBX8 to induce flowering. To test this hypothesis, the pull-down assay was used to evaluate whether the anti-His antibody could detect the CmBBX8-His protein compared to the glutathione (GST)-EV control when CmBBX7-GST protein was used as bait (Fig. 5a). The bimolecular fluorescence complementation (BiFC) assay was used to detect interactions between CmBBX7 and CmBBX8 in *N. benthamiana* leaves. First, the subcellular localization of CmBBX7 was investigated using transient expression in *N. benthamiana* leaves. *p35S::GFP* was used as a control, and green fluorescent protein (GFP) signals were observed in both the cytoplasm and nucleus of transiently transformed *N. benthamiana* leaf cells. When *CmBBX7* was fused to cauliflower mosaic virus 35S (*CaMV35S*) promoter-driven *GFP* and transiently expressed, the GFP signals overlapped with those of the nuclear marker D53-mCherry in transformed cells, suggesting that CmBBX7 may be localized in the nucleus (Supplementary Fig. S2), which is consistent with our previous results for CmBBX8 (Wang et al. 2020). pSPYNE-CmBBX7 and pSPYCE-CmBBX8 constructs were injected into *N. benthamiana* leaves via *Agrobacterium*-mediated transformation. Unlike the EV control, a yellow fluorescent signal appeared when pSPYNE-CmBBX7 was simultaneously present with pSPYCE-CmBBX8, and this signal completely overlapped with the red fluorescent signal of the nuclear marker (Fig. 5b). The interactions between CmBBX7 and CmBBX8 were further determined using the firefly luciferase complementation imaging (LCI) assay in *N. benthamiana*. The NLUC-CmBBX8 protein exhibited fluorescent signals when present simultaneously with CLUC-CmBBX7, but not with the EV (Fig. 5c). These results indicated that CmBBX7 interacts with CmBBX8 both in vivo and in vitro.

### CmBBX7 and CmBBX8 synergistically regulate *CmFTL1* expression

**Fig. 6:**
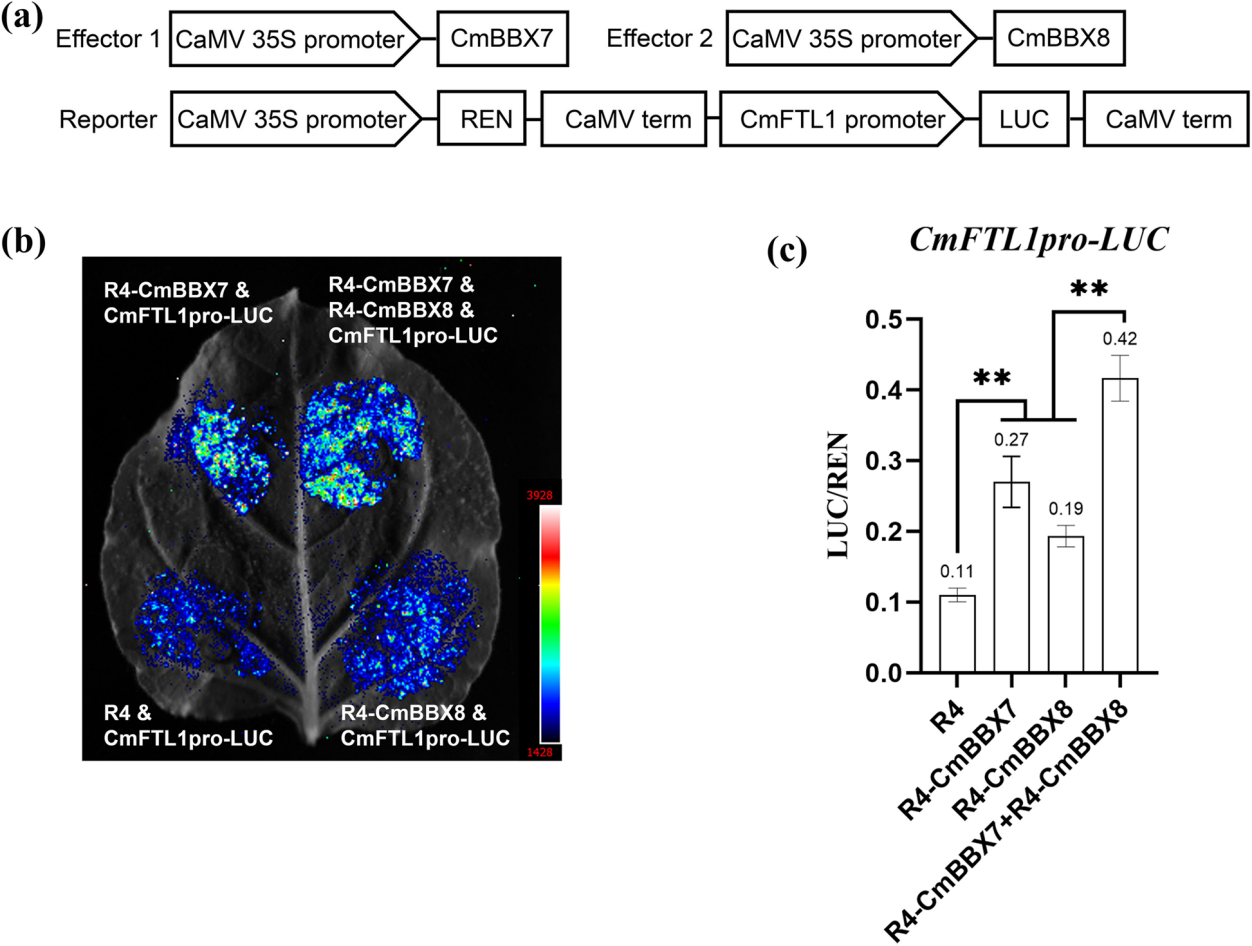
CmBBX7 and CmBBX8 synergistically regulate *CmFTL1* expression*.* **a** Effector and reporter vector construction diagrams for dual-luciferase assays. **b** Transient expression in *N. benthamiana* leaves of the *pCmFTL1::LUC* transgene. The graph shows the luminescence intensity. **c** The ratio of LUC to REN activity. Error bars indicate the standard deviations for six biological replicates. Significant differences are indicated with asterisks (**p < 0.01, ANOVA with Tukey’s post-hoc test)

To further explore the mechanism of the interaction between CmBBX7 and CmBBX8, the LUC assay was used to determine the effect of CmBBX7 and CmBBX8 interactions on the transcripts of *CmFTL1* based on a previously described method (Wang et al. 2021a, b, c). Compared with the EV control, both CmBBX7 and CmBBX8 could independently activate LUC driven by the *CmFTL1* promoter, and the activation effect of CmBBX7 was stronger than that of CmBBX8. When *CmBBX7* and *CmBBX8* were transfected into *N. benthamiana* leaves together, a stronger fluorescent signal was produced than that produced by either *CmBBX7* or *CmBBX8* alone (Fig. 6a, b).

To further substantiate the above results, chrysanthemum protoplasts were transfected using a previously described method (Higuchi et al. 2013). Compared with the EV, CmBBX7 or CmBBX8 could dramatically enhance the LUC/REN activity ratio, and the activation effect of CmBBX7 was more obvious than that of CmBBX8; it is noteworthy that the relative fluorescence activity was higher when both *CmBBX7* and *CmBBX8* were transfected together (Fig. 6c), which further indicated that CmBBX7 and CmBBX8 synergistically induce the expression of *CmFTL1* to promote flowering.

**Fig. 7:**
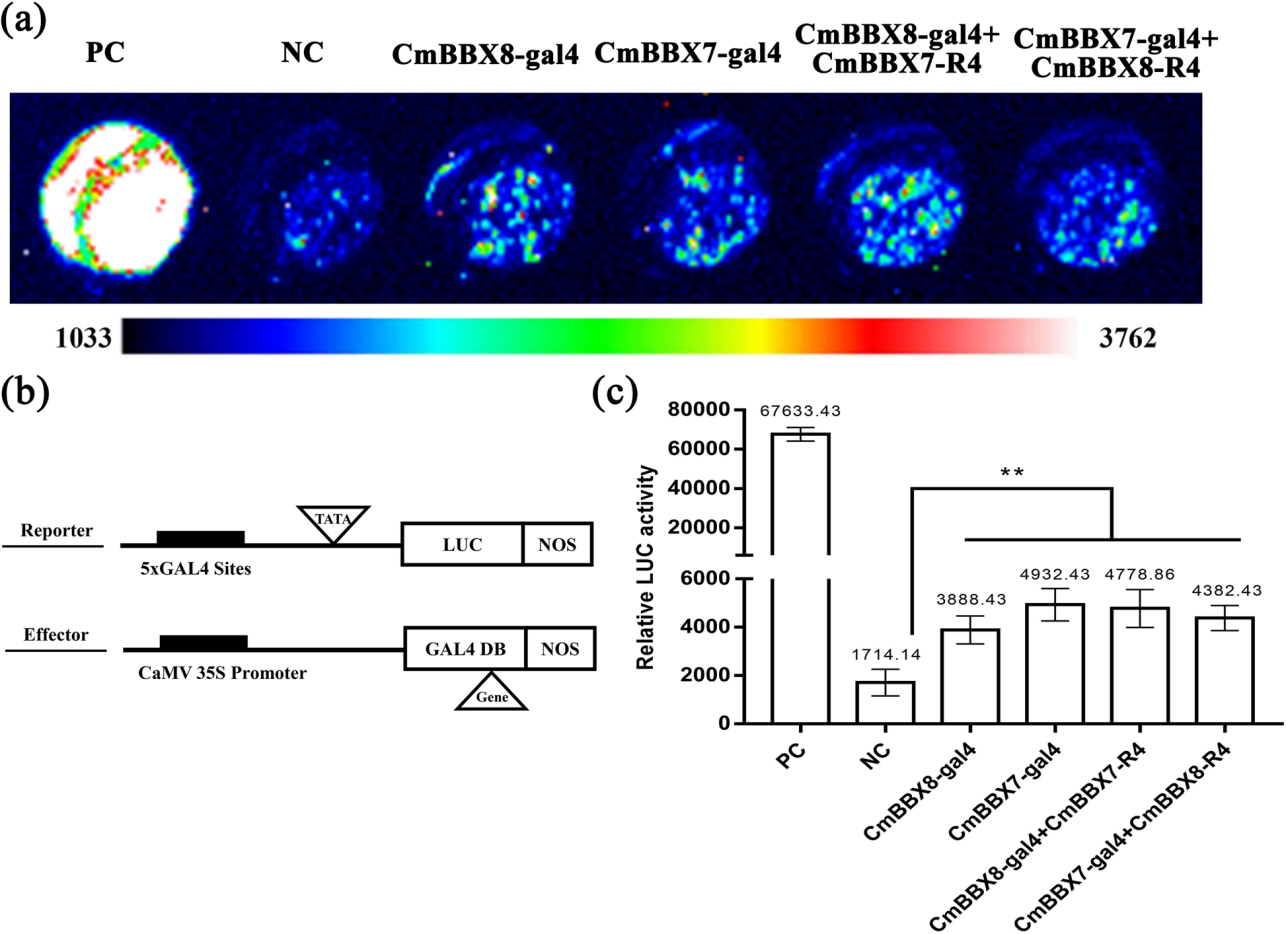
CmBBX7 and CmBBX8 do not mutually affect each other’s activity. Fluorescence images: from blue to red, the fluorescence value increases gradually. PC: positive control (AtARF5); NC: negative control (empty vector). **b** Vector construction diagrams for the reporter and effector constructs used in the transcription activity assay. The reporter construct contains five copies in tandem of the GAL4-binding sites upstream of the promoter (TATA), the firefly gene for luciferase, and Nos. The effector constructs contain the GAL4 DNA-binding domain (GAL4 DB) between the CaMV 35S promoter and Nos. **c** Relative LUC activity. Error bars indicate the standard deviations for six biological replicates. Significant differences are indicated with asterisks (**p < 0.01, ANOVA with Tukey’s post-hoc test)

To date, the mechanism by which CmBBX7 and CmBBX8 interact to promote the regulation of *CmFTL1* was unknown. To determine the underlying mechanism, a transcription activity assay was performed with the reporter and effector constructs (Fig. 7a), which contained five copies of GAL4-binding sites that were fused in tandem to drive the *LUC* gene, including the *CaMV35S* promoter, which drives the coding region of the *GAL4DB-CmBBX7/8* fusion. When the reporter and effector constructs were transfected into chrysanthemum protoplasts, the fluorescence intensity of CmBBX8 or CmBBX7 protein dramatically increased compared with that of the negative control (NC), indicating that CmBBX7 or CmBBX8 has transcriptional activation activity. However, the addition of *CmBBX7-R4* or *CmBBX8-R4* did not significantly enhance the transcriptional activation activities of *GAL4-CmBBX8* and *GAL4-CmBBX7*, respectively. This suggests that CmBBX7 and CmBBX8 may not affect each other’s transcriptional activation activity but may co-bind to the *CmFTL1* promoter to promote its expression (Fig. 7b, c).

## Discussion

In LD plants, Arabidopsis AtBBX7, a member of the group II BBX protein family, delays flowering through transcriptional repression of *CO* and *FT* (Cheng and Wang 2005). In SD plants, rice OsCOL9 and OsCOL10 have similar functions to their homologs BBX7 and BBX8, respectively, in Arabidopsis (Liu et al. 2016; Tan et al. 2017). The autumn-flowering chrysanthemum is an SD plant that has three FT-like genes: *FTL1, FTL2*, and *FTL3*. *FTL3* acts as a floral inducer and plays an important role under SD conditions (Oda et al. 2012); *FTL1* acts as an LD floral inducer, while the TERMINAL FLOWER 1 homologous gene *CsAFT* disrupts the FT–FD complex to delay flowering (Higuchi et al. 2013). The chrysanthemum BBX24 homolog CmBBX24, another member of the BBX family, was shown to play negative roles in flowering by inhibiting gibberellin (GA) biosynthesis (Yang et al. 2014). Moreover, the function of the chrysanthemum NF-YB8 homolog CmNF-YB8 in the acceleration of the transition from the juvenile to adult phase has been revealed (Wei et al. 2017). Through transcriptomic analysis, the photoperiod pathway involved in the regulation of flowering under SD conditions and the GA pathway under LD conditions were determined in the autumn-flowering chrysanthemum, respectively (Dong et al. 2017). In summer-flowering chrysanthemum, a day-neutral plant, the photoperiod and GA pathways under SD and the T6P and sugar signaling pathways involved in flowering under LD have been identified (Ren et al. 2016). In our previous report, we further revealed the function of CmBBX8 in the acceleration of flowering of the summer-flowering chrysanthemum cultivar ‘Yuuka’ (Wang et al. 2020). However, the regulation of flowering in day-neutral chrysanthemum is not well known. Thus, we performed the present detailed molecular study of CmBBX7, which showed that this protein interacts with CmBBX8 and accelerates flowering through direct regulation of *CmFTL1* via promoter binding in chrysanthemum cv. ‘Yuuka’.

The activation or inhibition of *FT* expression is not only regulated by a single transcription factor, but by multiple transcription factors under different environmental or hormonal stimulations. Indeed, various transcription factors synergistically regulate expression of the *FT* gene in *Arabidopsis thaliana*. The proximal Block A region of the *FT* promoter can be combined with the clock-regulated transcription factors CYCLING DOF FACTOR (CDF) and TEMPRANILLO (TEM) to inhibit *FT* transcription. The MADS transcription factors FLOWERING LOCUS C (FLC), SHORT VEGETATIVE PHASE (SVP), FLOWERING LOCUS M (FLM), and MADS AFFECTING FLOWERING (MAF) repress transcription by binding to the first intron of *FT* at low temperatures or before vernalization, whereas the AP2 transcription factors TARGET OF EAT 1 (TOE1), TARGET OF EAT 2 (TOE2), SCHLAFMUTZE (SMZ), and SCHNARCHZAPFEN (SNZ) repress transcription by binding close to the 3′ untranslated region in the photoperiodic flowering pathway (Golembeski and Imaizumi 2015).

The BBX protein family plays an essential role in the regulation of flowering. Many BBX transcription factors influence the flowering process by regulating the expression of florigen *FT*. *SlCOL4a* and *SlCOL4b* are potential flowering inducers in tomatoes that positively regulate flowering by promoting the expression of the *FT* homolog, *SFT* (Yang et al. 2020). *OsCOL10* (*OsBBX8*) suppresses flowering by reducing the expression of the FT homologs RICE FLOWERING LOCUS T 1 (RFT1) and Heading date 3a (Hd3a; Tan et al. 2017) in rice. However, BBX family transcription factors often synergistically regulate the expression of *FT*. BBX19 interacts with CO and may deplete CO activity, thereby inhibiting premature *FT* expression (Wang et al. 2014). RcCOL4 interacts with RcCO, thereby promoting RcCO binding to the *RcFT* promoter and activating its transcription (Lu et al. 2020). BBX17 interacts with CO and represses CO-regulated *FT* expression (Xu et al. 2022). At low temperatures, CO in *bbx28-bbx29* double mutant plants reduced the transcriptional activation of the *FT* promoter, thereby delaying flowering; however, there was no flowering-related phenotype in either the *bbx28* or *bbx29* single mutant (Wang et al. 2021a, b, c). Here, we showed that CmBBX7 interacts with CmBBX8 to regulate *CmFTL1* expression directly for flowering induction in chrysanthemum cv. ‘Yuuka’, and that the interaction may be important in allowing chrysanthemum to promote flowering promptly under photoperiod conditions. Collectively, these findings suggest that BBX transcription factors may require quantitative control during different seasons and under photoperiodic cues, thus allowing *FT* expression to be initiated.

It has been shown that *FT* levels are the result of a quantitative balance between promotion and inhibition; specifically, the quantitative balance between the activator CO and the deterrent TEM determines *FT* levels (Castillejo and Pelaz 2008). Before the flower-forming transition, *TEM*, *FT*, and *CO* expression can be detected in leaf cells with some spatial overlap in their distribution (Takada and Goto 2003). However, *TEM* levels are very low at this stage, which may not be sufficient to avoid CO from activating *FT*. This may be the general mechanism among LD plants to ensure strict regulation of flowering time (Castillejo and Pelaz 2008). We previously reported that CmRCD1 associates with CmBBX8 to delay flowering in summer chrysanthemum (Wang et al. 2021a, b, c). However, further studies are needed to determine whether CmRCD1 acts in a similar way to repress the activity of CmBBX7.

Collectively, our results suggest that CmBBX7 and CmBBX8 interactions could drive the expression of *CmFTL1*. This further implies that the previously reported driving activity of CmBBX8 on *CmFTL1* may have a dosage effect on multiple transcription factors that effectively interact with each other. It has been reported that under ethylene-induced conditions, the C-terminus of EIN2 and the EIN3 DNA-binding domain fuse to form dimers that interact with EIN3 to affect histone acetylation levels (Wang et al. 2021a, b, c). Therefore, we hypothesize that this interaction between CmBBX7 and CmBBX8 might form a heterodimer complex, which would increase transcriptional activation of the *CmFTL1* promoter; however, further in-depth studies are needed to investigate the detailed mechanism.

## Methods

### Plant materials and growing conditions for diurnal rhythm expression analysis

The cuttings of chrysanthemum cv. ‘Yuuka’ were obtained from the China Chrysanthemum Germplasm Resource Conservation Center of Nanjing Agricultural University (Jiangsu, China); planted in a mixture of nutrient soil, perlite, and vermiculite for rooting; and maintained under LD conditions (16-h light/8-h dark, constant temperature of 23 °C, and relative humidity of 40%) for 15 days. When the plants grew to have more than 14 leaves, they were transplanted to incubators set to a 16-h or 8-h photoperiod with a constant temperature of 23 °C and relative humidity of 40%.

### Vector construction and genetic transformation

To construct the genetic transformation vectors, we used the primer pair pORER4-CmBBX7-F/R to clone and insert the *CmBBX7* ORF into the *CaMV35S* promoter-driven overexpression vector pORE-R4. Pull-down assays were performed using a full-length *CmBBX7* fusion with a *GST* tag in the pGEX4T1 vector with primer pair pGEX-CmBBX7-F/CmBBX7-R and *CmBBX8-HIS* (Wang et al. 2020). Transcriptional activation of CmBBX7 assay was performed using vectors with primer pairs *pBD-CmBBX7*(*B-boxes*)F/R*, pBD-CmBBX7*(*∆-boxes*)F/R*, pBD-CmBBX7*(*CCT*)F/R*,* and *pBD-CmBBX7*(*∆CCT*)F/R*.* The yeast expression vectors pAD-CmBBX7 and pHIS2-CmFTL1pro were constructed for the Y1H assay. The BiFC assay was performed with the primer pair CmBBX7-NE-F/R containing BamHI and XhoI sites to amplify the *CmBBX7* sequence and insert the pSPYNE vector, which harbored the reporter gene encoding YFP, to obtain the pSPYCE-CmBBX8 construct, as previously described (Wang et al. 2021a, b, c). For the LCI assay, the pCLUC-CmBBX7-F/R and pNLUC-CmBBX8-F/R constructs containing *Bam*H I and *Sal* I sites were used to amplify the *CmBBX7 and CmBBX8* sequence, respectively, which were then inserted into the pLUC vector. GALDB4-CmBBX7 was constructed for the transient activation of protoplasts using the LR recombination method. The primer sequences for these constructs are listed in Supplementary Table S1.

The chrysanthemum was genetically transformed as described by Simmons et al. (2009). For the LUC assay, the *CmFTL1-pro* sequence was cloned into the pGreenII 0800-LUC vector by pGreenII 0800-LUC-CmFTL1-pro-F/R, containing a fluorescent group.

### Yeast hybrid experiments

The Y187 strain was prepared for the Y1H assay, we mixed pAD-CmBBX7 and pAD-GUS with pHIS2-CmFTL1pro respectively, and transformed them into Y187 strain using the lithium acetate method.Then inoculated on SD/His-Trp-Leu-plates, and screened for the lowest concentration range of 3-AT that was sufficient to inhibit the growth of pHis2-CmFTL1pro, which would be used for co-transformation screening. The plates were incubated at 30 °C and photographed for recording for approximately two to three days.

### BiFC assay and luciferase imaging

The constructed vector plasmid was transformed into *A. tumefaciens* strain EHA105 or GV3101, which was further incubated until the optical density at 600 nm (OD_600_) reached approximately 1.0. Subsequently, 6 mL of bacterial solution was centrifuged at 5000 rpm for 10 min and washed once with an equal volume of the solution (0.5 M MES, 200 μL; 1 M MgCl_2_, 100 μL; 100 mM phenylbutyrate, 100 μL). Thereafter, the pellets were resuspended and washed once with an equal volume of treatment solution, and 5–8 mL of the solution (0.5 M MES, 200 μL; 1 M MgCl_2_, 100 μL; 100 mM acetosyringone, 10 μL; supplemented with redistilled water to 10 mL) was added and resuspended. The injection solution for each combination was adjusted to obtain the same OD_600_ of 0.5, incubated in the dark at 25 °C for approximately 3 h, and injected into healthy *N. benthamiana* leaves. The injected *N. benthamiana* plants were incubated in the dark for 24 h, followed by 48 h in the light, and the fluorescence signal was observed and photographed under a laser confocal microscope (ZEISS, LSM780) using a previously described method (Lai et al. 2013). Fluorescence activity detection was performed using a CCD camera (NightOWL818 II LB983) and IndiGO software according to a published method (Kost et al. 1995).

### Pull-down assay

Based on the method reported by Wang et al. (2021a, b, c), the combination of CmBBX7-GST and CmBBX8-HIS or GST-empty and CmBBX8-HIS was incubated for 2 h at 4 °C after protein induction, and pre-washed GST magnetic beads (Promega, Madison, WI, USA) were added and incubated overnight at 4 °C. The beads were then washed at least three times with wash buffer to remove the heteroproteins, the GST-tagged proteins bound to the magnetic beads were eluted using reduced GST, and the eluted proteins were used for measuring protein–protein interactions by western blotting with anti-His antibody (Thermo Fisher, Waltham, MA, USA), as reported previously (Francisco-Velilla et al. 2016).

### Transient activation experiments in protoplasts

Protoplasts were prepared using the young spreading leaves of 3–4-week-old seedlings of chrysanthemum cv. ‘Yuuka’ following a previously described method (Higuchi et al. 2013). Briefly, the leaf strips were immersed in an enzymolysis solution for 2–3 h at 28℃ with shaking and 50 rpm. Thereafter, the nylon membrane was washed with W5 Buffer, and the protoplasts were filtered and centrifuged; 1 mL of MMg buffer was added to the pellets and mixed gently in an ice bath for 30 min. The PEG-mediated method was then used to transform the plasmid. The transformed protoplasts were incubated for more than 16 h. Fluorescein sodium was added, the solution was incubated in the dark for 15 min, and the reaction was measured using a GLOMAX® -20/20 instrument to determine the REN and LUC activities, respectively; the ratio of LUC/REN was calculated.

### qRT-PCR analysis

Total RNA was isolated from the leaves of chrysanthemum seedlings grown at 23℃ for 4 weeks using a Rapid RNA Isolation Kit (Huayueyang, Beijing, China) according to the manufacturer’s instructions. cDNA was synthesized by reverse transcription of the extracted leaf RNA using the PrimeScript RT reagent kit (Takara, Beijing, China). Real-time PCR analysis was then performed mixing the 0.5 μL cDNA, 10 μL SYBR GREEN and 7 μL H2O as a template on a Roche LightCycler 480 II system with the SYBR Premix ExTaq II kit (Takara, Dalian, China) and the following primer pairs: qRT-CmBBX7-F/qRT-CmBBX7-R and qRT-CmFTL1-F/qRT-CmFTL1-R (Supplementary Table S2). The qRT-PCR procedure is 95℃/2 min, followed by 40 cycles of 95℃/15 s, 60℃/15 s and 72℃/15 s. *CmEF1α* was used as the reference gene. Three biological and three technical replicates were performed for each sample. The relative expression levels of the corresponding genes were calculated using the 2^–ΔΔCT^ method according to the cycle threshold (Ct) value (Livak and Schmittgen 2001).

### EMSA

EMSA was performed using a LightShift Chemiluminescent EMSA Kit (Thermo Fisher, New York, NY, USA). The probe sequences used for EMSA were synthesized as shown in Supplementary Table S3 and labeled using the Beyotime Probe Labelling Kit. The purified proteins and labeled probes were prepared according to the instructions provided in the EMSA kit, incubated for 30 min at 25℃, and the reaction was terminated by adding 2 μL of blue loading buffer. Polyacrylamide gel electrophoresis was performed, followed by constant-flow wet transfer of the membrane for more than 2 h, ultraviolet cross-linking (2000 J for 5 min), blocking, and washing of the nylon membrane. Finally, a CCD camera was used to observe and analyze the chemiluminescence signal.

### ChIP-qPCR

The leaves of p35S::GFP-CmBBX7 and EV (negative control)-transfected plants were selected as experimental materials for cross-linking. Chromatin was extracted from the leaves, interrupted, and then A/G magnetic beads and GFP antibody (both from Thermo Fisher Scientific) were added. Finally, the DNA fragments were examined by qRT-PCR as described above with the P1-P5 primer.

### Statistical analyses

Analysis of variance (ANOVA) with Tukey’s honest significant difference post-hoc test was used to determine statistical significance. Differences in all qPCR data were considered significant at *p* < 0.01.

### Supplementary Information

**Additional file 1: Supplementary Table S1.** Primer sequences for cloning. **Supplementary Table S2.** Primer sequences for vector. **Supplementary Table S3.** Primer sequences for qRT-PCR. **Supplementary Table S4.** Primer sequences for EMSA.

**Additional file 2: Supplementary Figure S1.** Transcriptional activation of CmBBX7.

**Additional file 3: Supplementary Figure S2.** Subcellular localization of CmBBX7.

## Data Availability

The authors confirm that all data in this study are included in this published article (and its supplementary information file).

## Abbreviations

3-AT: Amino-1, 2, 4-triazole
BIFC: Bimolecular fluorescence complementation
CDF: CYCLING DOF FACTOR
CO: CONSTANS
EMSA: Electrophoretic mobility shift assay
EV: Empty vector
FLC: FLOWERING LOCUS C
FLM: FLOWERING LOCUS M
FT: FLOWERING LOCUS T
GA: Gibberellin
GFP: Green fluorescent protein
Hd3a: Heading date 3a
HSD: Honestly significant difference
LCI: Luciferase complementation imaging
LD: Long day
LUC: Luciferase
MAF: MADS AFFECTING FLOWERING
NC: Negative control
OE: Overexpression
ORF: Open reading frame
REN: Renilla luciferase
RFT1: RICE FLOWERING LOCUS T 1
SD: Short day
SMZ: SCHLAFMUTZE
SNZ: SCHNARCHZAPFEN
SOC1: SUPPRESSOR OF OVEREXPRESSION OF CONSTANS 1
SVP: SHORT VEGETATIVE PHASE
TEM: TEMPRANILLO
TOE1: TARGET OF EAT 1
TOE2: TARGET OF EAT 2
WT: Wild-type
Y1H: Yeast single hybridization
