# Supplementary material for: BBX7 interacts with BBX8 to accelerate flowering in chrysanthemum
Source: Mol Hortic. 2023 Apr 1;3:7. doi: 10.1186/s43897-023-00055-2 (PMC10515231; doi:10.1186/s43897-023-00055-2)
Supplement: Supplementary file 1 — Additional file 1: Supplementary Table S1. Primer sequences for cloning. Supplementary Table S2. Primer sequences for vector. Supplementary Table S3. Primer sequences for qRT-PCR. Supplementary Table S4. Primer sequences for EMSA. [file 43897_2023_55_MOESM1_ESM.docx]

Table. S1 Primer sequences for cloning

*CmBBX7-F*

ATGGGTTACAATTGTGATTACTGTG

Sequences (5’-3’)

Primes name

*CmBBX7-R*

TCAGAAACTTGTTGCTTCATTCATG

*CmFTL1-PRO-F*

AAGACAGCTCACTGAGTGACTATGC

*CmFTL1-PRO-R*

TGAAGTTTGCTTCTTTCAAGCGTGT

Table. S2 Primer sequences for vector

GGCCGAATTCATGGGTTACAATTGTGATTA

Sequences (5’-3’)

Primes name

CGACGGATCCTCAGAAACTTGTTGCTTCATTCA

*pBD*-*CmBBX7-R*

*pBD*-*CmBBX7-*F

GGCCGAATTCATGGGTTACAATTGTGATTA

*pBD*-*CmBBX7-BBXs*-F

CGACGGATCCTCACAAAGTCTGTCGACTATGTG

*pBD*-*CmBBX7-BBXs*-R

GGCCGAATTCAATTGTTATTCTGGATGCCC

CGACGGATCCTCAGAAACTTGTTGCTTCATTCA

*pBD*-*CmBBX7-∆BBXs*-R

*pBD*-*CmBBX7-∆BBXs*-F

GGCCGAATTCAGAAGTGATGCTGTTCTTCG

*pBD*-*CmBBX7*-CCT-F

CGACGGATCCTCAGAAACTTGTTGCTTCATTCA

GGCCGAATTCATGGGTTACAATTGTGATTA

*pBD-CmBBX7*-∆CCT-F

*pBD*-*CmBBX7*-CCT-R

CGACGGATCCTCAGATGGTAGTGCTTCCAGGAC

*pBD-CmBBX7*-∆CCT-R

tggcgcgccactagtggatccATGGGTTACAATTGTGATTACTGTGG

*pSPYNE*-*CmBBX7*-F

acatcccgggagcggtaccctcgagGAAACTTGTTGCTTCATTCATGGG

CGGGATCCAATGGGTTACAATTGTGATTA

*pSPYNE*-*CmBBX7*-R

*pORE-R4*-*CmBBX7*-F

CGGAATTCTTGAATGAAGCAACAAGTTTC

*pORE-R4*-*CmBBX7*-R

CCGGAATTCATGGGTTACAATTGTGATTA

CCGGAATTCATGGGTTACAATTGTGATTA

*pGEX*-*CmBBX7*-F

CGGAATTCAAGACAGCTCACTGAGTGACTATG

CCGCTCGAGGAAACTTGTTGCTTCATTCA

A

*Phis2*-*CmFTL1pro*-F

*pGEX*-*CmBBX7*-R

CGACGCGTAATGAAGTTTGCTTCTTTCAAGT

*Phis2*-*CmFTL1pro*-R

CCGGAATTCATGGGTTACAATTGTGATTA

*pAD*-*CmBBX7-*F

CGCGGATCCCGAAACTTGTTGCTTCATTCA

*pAD*-*CmBBX7-R*

*pCLUC*-*CmBBX7-F*

cggggcggtacctccgaatccATGGGTTACAATTGTGATTACTGTGG

*pCLUC*-*CmBBX7-R*

acgaaagctctgcaggtcgacTCAGAAACTTGTTGCTTCATTCAT

*pNLUC*-*CmBBX8-F*

gagctcggtacctccggatccATGGGTTATAATTGTGATTATTGCC

*pNLUC*-*CmBBX8-R*

cgcgtacgagatctggtcgacGTAGCTTCTAGTTTGGCTCATGGGA

CGGGATCCAAGACAGCTCACTGAGTGA

*pGreenII -0800-LUC-CmFTLI-pro-F*

GGACTAGTTGAAGTTTGCTTCTTTCAAG

*pGreenII -0800-LUC-CmFTLI-pro-R*

Table. S3 Primer sequences for qRT-PCR

Sequences (5’-3’)

Primes name

GCTTACTTGTGTTTATCGTG

*qRT-CmBBX7-F*

TGCCTTTTCATCCACACATC

*qRT-CmBBX7-R*

TGCCTTTTCATCCACACATC

CTGGCTGGTAATGATTCAAATTGTG

*qRT-CmBBX8-F*

TGCCTTTTCATCCACACATC

CAACAGAAGTATCTTTAGCACCAAAC

*qRT-CmBBX8-R*

TGCCTTTTCATCCACACATC

AATCGTGTGCTATGAGAGCC

*qRT-CmFTL1-F*

TGCCTTTTCATCCACACATC

GCTTGTAACGTCCTCTTCATGC

*qRT-CmFTL1-R*

TGCCTTTTCATCCACACATC

*CmEF1α-F*

TGCCTTTTCATCCACACATC

*CmEF1α-R* TGCCTTTTCATCCACACATC

TTTTGGTATCTGGTCCTGGAG

*P1-F*

TGCCTTTTCATCCACACATC

CCATTCAAGCGACAGACTCA

*CORE-F*

GAGAATATGTTTGTACTTGAGG

TCGTGTTGAAAACTGGAAACATG

*CORE-F*

GAGAATATGTTTGTACTTGAGG

*P1-R*

TGCCTTTTCATCCACACATC

GTTAGCAAGATTACGATGTC

*CORE-F*

GAGAATATGTTTGTACTTGAGG

*P2-F*

TGCCTTTTCATCCACACATC

TCTCAACATAGTAACGGTAC

TCTC

*CORE-F*

GAGAATATGTTTGTACTTGAGG

*P3-F*

TGCCTTTTCATCCACACATC

*P2-R*

TGCCTTTTCATCCACACATC

ATGTATTCCTTCTAGTTCGG

*CORE-F*

GAGAATATGTTTGTACTTGAGG

AGAGCAGTATTTTGTAACGG

*CORE-F*

GAGAATATGTTTGTACTTGAGG

*P3-R*

TGCCTTTTCATCCACACATC

CTTGTCAGATGGGTATGACG

GAGAATATGTTTGTACTTGAGG

*P4-R*

TGCCTTTTCATCCACACATC

*P4-F*

TGCCTTTTCATCCACACATC

GCTTCTTTCAAGCGTGTTGG

TTTGTACTTGAGGTGTTTGG

*P5-F*

TGCCTTTTCATCCACACATC

ATTGTGATTACTGTGGTGAG

TTCTCATGTGGCGTTTGGAT

*P5-R*

TGCCTTTTCATCCACACATC

Table. S4 Primer sequences for EMSA

GAGAATATGTTTGTACTTGAGGTGTTTTGTGGTGCCACATGCT

ATG

Primes name

Sequences (5’-3’)

CATAGCATGTGGCACCACAAAACACCTCAAGTACAAACATATTCTC

*CORE-F*

TGCCTTTTCATCCACACATC

*CORE-R*

TGCCTTTTCATCCACACATC

*TG-box-F*

TGCCTTTTCATCCACACATC

ATAAATACGTTAACAAATATCACGTTTTGGAGTTAACAAATTTCTC

*TG-box-R*

TGCCTTTTCATCCACACATC

GAGAAATTTGTTAACTCCAAAACGTGATATTTGTTAACGTATTTAT
