# Supplementary figures and images for: BBX7 interacts with BBX8 to accelerate flowering in chrysanthemum
Source: Mol Hortic. 2023 Apr 1;3:7. doi: 10.1186/s43897-023-00055-2 (PMC10515231; doi:10.1186/s43897-023-00055-2)

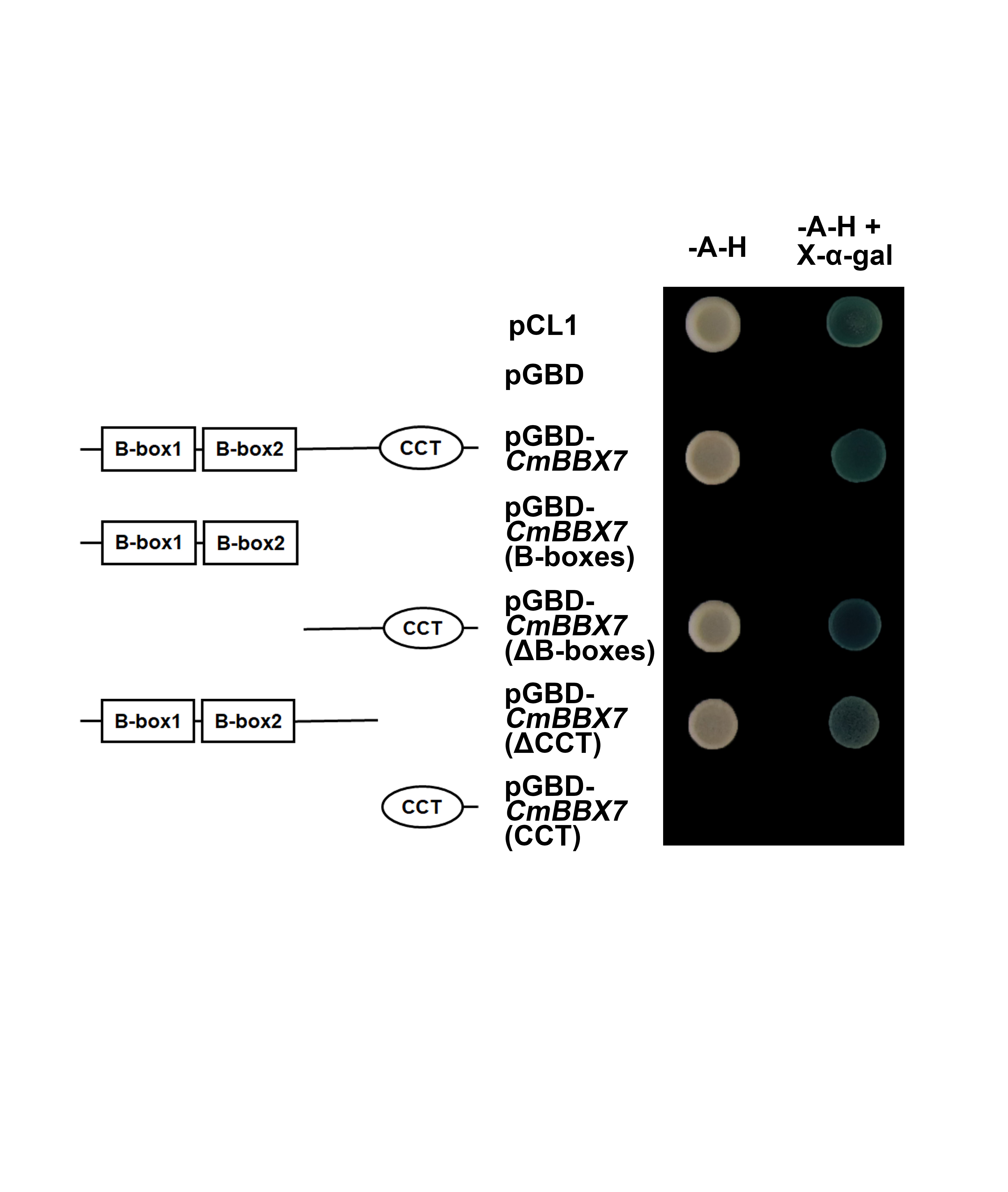

Supplement: Supplementary file 2 — Additional file 2: Supplementary Figure S1. Transcriptional activation of CmBBX7. [file 43897_2023_55_MOESM2_ESM.jpg]

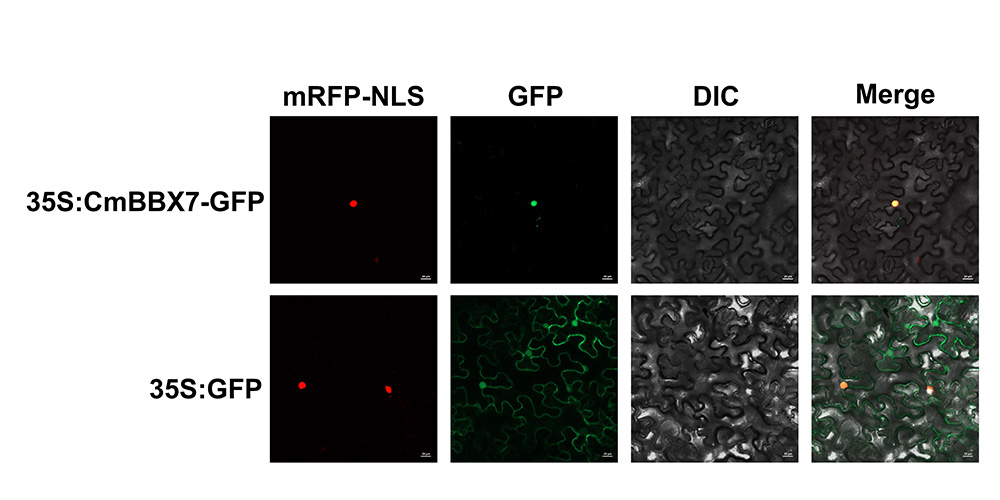

Supplement: Supplementary file 3 — Additional file 3: Supplementary Figure S2. Subcellular localization of CmBBX7. [file 43897_2023_55_MOESM3_ESM.jpg]
